# The Mediating Role of Perceived Social Support in the Relationships Between General Causality Orientations and Locus of Control With Psychopathological Symptoms

**DOI:** 10.5964/ejop.v14i3.1563

**Published:** 2018-08-31

**Authors:** İhsan Dağ, Gamze Şen

**Affiliations:** a Department of Clinical Psychology, Hacettepe University, Ankara, Turkey; Department of Psychology, Webster University Geneva, Geneva, Switzerland; University of Western Ontario, London, Canada

**Keywords:** locus of control, perceived social support, psychological symptoms, general causality orientations, OCD, depression

## Abstract

The main aim of this study is to investigate the mediator role of perceived social support in the relationship between general causality orientations and locus of control with psychopathological symptoms. Total 751 participants were consisted of 558 female ages between 17 and 36 (Female M = 19.03, SD = 0.09) (74.3%), 192 male ages between 17 and 37 (Male M = 20.71, SD = 0.17) (25.6%) and a participant who did not provide any gender information. We used the General Causality Orientations Scale (GCOS) and Locus of Control Scale (LOCS) in order to understand the basic motivation for the emergence of behavior. Beck depression Inventory (BDI) used to evaluate the psychological symptoms for depression, Maudsley Obsessive Compulsive Inventory (MOCI) for obsessive-compulsive symptomology and Brief Symptom Inventory (BSI) for overall psychological distress and finally to evaluate mediating role of social support used the Perceived Social Support Scale (PSSS). According to the results, having internal locus of control and autonomy orientation have shown positive effect to statistically significant predictors for psychological symptomology, having external locus of control and impersonal orientation have shown negative effect. Perceived social support was found to be suited for the role of partial mediator, and social support from friends was found to have more positive roles than social support from family. In conclusion, exceedingly considerable to conduct further research in order to contribute to the understanding of the mediating role of general causality orientations and locus of control with psychopathology symptomology.

Causality orientations, as proposed in Self Determination Theory (SDT), differ from Rotter’s locus of control in respect to their emphasis on the causality of behaviors. Locus of control, which was proposed by Rotter (1966) and implemented in social learning models, is one of the prominent concepts in understanding the cognitive processes of behavior (Rotter, 1966; Skinner, 1996). According to the locus of control concept, individuals are motivated to behave in a specific pattern when they feel that pattern of behaviors will be subsequently reinforced. When these individuals acknowledge the positive or negative outcomes of self-behaviors, they tend to expect similar outcomes from similar behaviors in advance, and so these behaviors become generalized. Consequently, individuals might gain a set of expectations (belief systems) about the origin of reinforcements, which are either dependent solely on themselves or are external in origin, such as chance, fate, or other external forces. While the first situation is considered as an internal locus of control, the second situation is taken considered as representing an external locus of control.

As the appearance of locus of control coincides with self-determination theory, Heider (1958) and deCharms (1968) described the source of behaviors’ as being internal, external, and impersonal. In the following years, Deci and Ryan (1985) studied these terms along with causality orientations, describing them as autonomous, control, and impersonal, respectively. General causality orientations are considered as a set of concepts accounting not only internal processes, but also external processes and motivational states. Individuals who follow autonomous orientations initiate or modify their behavior according to their own motives and objectives. These individuals behave through their own choice and tend to acknowledge the origin of their behaviors as the self. The control orientation can be initiated or modified, externally like awarding or internally as “I should do” or “this should be done”. While these individuals seek to find external control, they also acknowledge their surrounding environment as controlling. Conversely, in impersonal orientations, initiation and modification of behaviors are under the control of another individual. These individuals do not possess a set of beliefs allowing them to think that they can accomplish objectives and goals by themselves; their behaviors are defined as non-motivated and non-determined (Deci & Ryan, 1985).

It is important to point out the distinction between the causality orientations of self-determination theory and the locus of control concept proposed by Rotter (1966). According to locus of control, the source of behavior is dependent on the pattern of reinforcement (Rotter, 1966, 1975, 1990). On the other hand, deCharms (1968) proposed that either the source is internal cognitive processes and personal choices, or external pressure or force are the determining factors. In short, while Rotter (1966) focused on the outcome of behavior deCharms pointed out the importance of priming factors on behaviors. There are several publications in the literature about the importance of locus of control within psychopathologies (Baydoğan & Dağ, 2008; Cheng et al., 2013; Dağ, 1992; Gülüm & Dağ, 2014). However, there are few studies about the priming factors of behavior and their relation to psychological symptoms (Brockelman, 2009; McGregor, McAdams, & Little, 2006). Studying the origin of behaviors through a combination of both viewpoints (locus of control and causality orientations) which provide different explanations is very important in broadening current understanding and increasing clarification.

Several important studies point out the importance of the genotype-environment relationship with respect to psychological symptoms. Despite similar environments, behaviors have been shown to vary greatly among individuals. As the behavioral tendencies and motivational states of individuals who experience similar environments cannot be assessed without an internal and external concept of control (Deci & Ryan, 1985, 2000; McAdams & Olson, 2010; Pelletier et al., 1999; Ryan, Deci, & Grolnick, 1995), the environmental impacts on psychological symptoms, such as positive and negative social support, cannot be separated from the personal concept of control, or motivational states (Deci & Ryan, 1985; Yıldırım, 2004a). In this respect, we have acknowledged social support as variable in order to understand the perception of environment in our study.

One purpose of every society is to convey its thought system and values to young people (Yörükoğlu, 1992). Although the expected behavior patterns of young people are evident in every culture, these expectations may differ due to differences between each culture (Akbağ, Sayıner, & Sözen, 2005). The young people involved in our study are thought to be shaped by their culture, and their perceptions of themselves and their environment are shaped the societal expectations that they grew up with and the environment in which they current live. In this sense, Turkey has a rich cultural structure in terms of shaping young people’s perception of social support. Procidano and Heller (1983) defined social support as an individual’s own subjective judgment about whether they have enough social support to function. Social support has been studied extensively from different perspectives (Caplan, 1973; Lewin, 1951). In following studies, psychological symptoms and well-being have been investigated with regard to social support, and social support has been found to significantly decrease psychological symptoms (Wight et al., 2006; Yıldırım, 2004a). Self Determination Theory also proposes that with social support, personal well-being is expected to improve (Deci & Ryan, 1985). However, the tendency to show psychological symptoms in individuals with different personalities has not been studied so far in respect to role of social support. Within this framework, the perception of social support is going to be studied in relation to psychological symptoms with general causality orientations and locus of control. Until now, discussion of the distinctions and similarities between general causality orientations and locus of control has been very limited in the literature. In this study, while the distinction between causality orientations and locus of control has been emphasized, the relationship of these concepts with psychological symptoms is going to be investigated, with social support as a mediating factor. Moreover, clarification of the role of social support — acknowledged as external factor — is thought to be an important mediator between the tendency for psychological symptoms with locus of control and general causality orientations in which motivational state and internal/external control which lie in the base of both locus of control and causality orientations.

Turkey is known as comprising both Western (personal) and Eastern (community) psycho-cultural values (Kağıtçıbaşı, 2005; Sayın, 1987). Owing to the socio-cultural diversity of Turkey, getting data from different region of the country will provide breadth of understanding in cultural aspect. The period of adolescence is the period when the perception of social support goes from family to friend. It is important for the adolescents to support from both family and friends in Turkish culture (Yörükoğlu, 1992). For this reason, our study has been conducted with participants who have were bachelor students with an age range of 17 to 37 in order to best observe the effect of social support. In this study, participants are assessed for emerging psychological symptoms along with general causality orientations and locus of control, with social support being a partial mediator. In order to expand the scope of study and investigate different cultural backgrounds, students from 10 different Turkish Universities have been incorporated into the study.

## Methods

### Participants

To conduct this research, 799 undergraduate students (568 Female, 227 Male, 4 did not want to specify gender) were recruited. Participants in different Universities were reached by random assignment and participation in the research is voluntary. Permission has already been granted for the application from these universities. The presence of the participant's reported psychiatric diagnosis is the only criterion for exclusion. The data of 16 participants were not included in the study, because they had a psychiatric diagnosis and were on medication. Once the rest of the data is obtained, the homogeneity of the variances and normal distributions from the basic assumptions of the statistic was tested. Subsequently, the data of the participants who were outside the normal distribution were examined. It was found that these participants responded randomly or left blank to an unacceptable level. According to results of the analysis therefore, the data of 32 participants outside the normal distribution were removed from the study, resulting in a final sample of 751 comprising 558 females (74.3%), 192 males (25.6%), and 1 participant with unknown gender. The demographic distribution of participants is shown in Table 1a and Table 1b.

**Table 1a:** Sample Distribution of Age, and Gender

Gender	*N*	Minimum age	Maximum age	*M*	*SD*
Female	558	17	36	19.93	2.32
Male	192	17	37	20.71	2.38
Total	750	17	37	20.14	2.36

**Table 1b:** Sample Distribution of University

University	Region	Participants	Percent
Erzincan University	Eastern Anatolia	31	4.1
Gazi University	Central Anatolia	89	11.9
İzmir University	Aegean Region of Anatolia	100	13.3
Konya University	Central Anatolia	76	10.1
Çukurova University	Mediterranean region of Anatolia	33	4.4
İstanbul Bilim University	Marmara Region of Anatolia	100	13.3
Antalya University	Mediterranean region of Anatolia	92	12.3
Ufuk University	Marmara Region of Anatolia	46	6.1
Dicle University	Southeastern Region of Anatolia	87	11.6
Ondokuzmayıs University	Black sea Region of Anatolia	97	12.9
Total		751	100.0

### Measures and Materials

*General Causality Orientation Scale (GCOS;* *Deci & Ryan, 1985**):* Autonomy, control, and impersonal orientations were measured by the GCOS (Deci & Ryan, 1985); a Turkish adaptation of the scale was developed by Şen and Dağ (2016). The original GCOS consisted of 3 subscales and 40 items which comprise autonomous orientations (16 items), impersonal orientations (14 items), and control orientations (10 items). Each item of the GCOS is rated on a 7-point scale. The reliability and validity of the GCOS has been demonstrated (Şen & Dağ, 2016). In this study, Cronbach’s alpha values are .86 for autonomy, .71 for control and, .76 for impersonal; the test-retest coefficient throughout a 3-week period was .81 for the total scale. The total variance of the entire scale is reported as 32.4%.

*Locus of Control Scale (LOCS;* *Rotter, 1966**):* Following the pioneering works of Phares (1957) and James (1957), Rotter (1966) developed the scale with the aim of investigating the perspective (interior-exterior) of individuals toward generalized control expectations. Moreover, the scale is established in way that it enables the measurement of general expectations or beliefs, to test whether reinforcements are under control of external (chance, fate) or internal forces. The Turkish adaptation of the Locus of Control Scale, developed by Dağ (2002), consisted of 5-point Likert-type scale with a total of 47 items. Higher total scores relates to the increase in external locus of control. The Turkish version of the scale had a Cronbach alpha coefficient of .92, and a monthly interval test-retest reliability coefficient of .88 (Dağ, 2002). In this study the Cronbach’s alpha is .90 for the total scale.

*Beck Depression Inventory (BDI;* *Beck, Ward, Mendelson, Mock, & Erbaugh, 1961**):* The original scale was developed (Beck et al., 1961), and is a widely used self-report tool for assessing depressive symptoms; it consists of 21 items, each of which is rated on a 4-point scale. The aim of this scale not to diagnose depression, but rather to identify the symptoms of depression and their severity. The Turkish adaptation of the scale was developed by Hisli (1988, 1989). Turkish adaptation version was used for this research. Higher scores on the BDI represent the greater severity of depressive symptoms. Arkar and Şafak (2004) reported a Cronbach’s alpha value of .90 for the Turkish adaptation of the BDI. In this study the total scale had a Cronbach’s alpha value of .89.

*Perceived Social Support Scale (PSSS;* *Procidano & Heller, 1983**):* The original scale was developed by Procidano and Heller (1983) and aimed to measure the level of perceived social support from friends, family, and teachers. The Turkish adaptation of PSSS was developed by Yıldırım (1997) and consists of a total of 50 items. Only the family and friends’ aspects are used as a part of this research. Responses are recorded in a triple grading format (suits me = 1, partially suitable = 2, not suitable for me = 3). Cronbach’s alpha values of total PSSS, the friends and family aspects .93, .91, and .93 respectively; test-retest reliability of these were .89, .86, and .86 (Yıldırım, 2004b). In this study the Cronbach’s alpha for the total scale was .93.

*Maudsley Obsessive Compulsive Inventory (MOCI;* *Sanavio & Vidotto, 1985**):* The MOCI was used to investigate obsessive compulsive symptoms. The instrument was found to reliably discriminate between obsessional patients and normal controls, and between patients with anorexia nervosa versus those with anxiety disorders. The Turkish adaptation of the scale was developed by Erol and Savaşır (1988). The MOCI contains 37 items in a true-false format. The MOCI is a form of self-report scale, participants fill out themselves (Sanavio & Vidotto, 1985). Higherscores indicate a rise in obsessive-compulsive symptoms. In this study the Cronbach’s alpha value for the total scale was .81.

*Brief Symptom Inventory (BSI;* *Derogatis, 1992**):* The BSI is an instrument that evaluates psychological distress and psychiatric disorders. The BSI is a 53-item self-report scale that uses a 5 point Likert scale (Derogatis, 1992; Şahin & Durak, 1994). Rising scores reflect an increase in symptom severity. The Turkish version of the BSI was found to consist of 5 factors during psychometric analysis: “Anxiety,” “Depression,” “Negative self,” “Somatization,” and “Hostility.” The Cronbach's alpha values of these factors were .87, .88, .87, .75, and .76 respectively.

### Procedure

This study, which is a part of project founded by The Scientific And Technological Research Council Of Turkey (TUBITAK) (Project no.114K086), attained the necessary permissions from the Hacettepe University Ethics Commission. The aim of the main study is to determine the variables affecting the psychological well-being of the individuals at the university level and to test a model that assesses variables that have positive intermediary role. Owing to the total size of the results, the findings that include part of the study are presented in this research paper.

After obtaining written permission from the participating universities, questionnaire booklets were administered to participants during class by researchers. Booklets took approximately 45-50 minutes to complete. The scales were listed in a different order to remove the order effect. Participants were informed in writing about the study and written consent was obtained. When the data of the study were analyzed, the correlation values between the scales and the subscales were included in addition to the mean, standard deviation and standard error values of the variables. Finally, path analysis was conducted and the model was tested via structural equation modeling. Data was analyzed using SPSS 20.0 and AMOS 23.0 software.

## Results

Firstly, the data was examined to check the basic assumptions of multivariate statistics, namely: normality, linearity, and homogeneity of variance. Once these assumptions had been verified, data analysis progressed to the next stage. Descriptive statistics for male and female subscale, PSSS, LOCS, and GCOS are shown in Table 2.

**Table 2:** Descriptive Statistics for Male and Female Subscale, PSSS, LOCS, and GCOS

Variable	Female (558)			Male (192)		
	*M*	*SD*	*SE*	*M*	*SD*	*SE*
GCOS-AUTONOMY	90.59	11.51	0.48	83.85	14.47	0.41
GCOS-CONTROL	42.97	8.07	0.34	43.19	7.05	0.50
GCOS-IMPERSONAL	45.74	11.16	0.47	46.31	10.63	0.76
LOCS- INTERNAL	75.28	11.54	0.46	79.90	12.96	0.43
LOCS- EXTERNAL	46.91	9.91	0.43	45.91	10.51	0.51
LOCS-TOTAL	122.34	16.10	0.68	120.57	17.42	0.57
PSSS	42.29	8.36	0.36	45.92	11.10	0.80
PSSS-FRIENDS	16.12	3.80	0.16	18.08	5.12	0.37
PSSS-FAMILY	26.21	6.38	0.27	27.88	7.50	0.54
BDI	12.07	9.27	0.39	12.34	9.68	0.69
MOCI	14.93	5.61	0.23	13.74	5.81	0.42
BSI	57.83	34.93	0.65	58.20	31.65	0.53

*Note.* GCOS = General Causality Orientations Scales; LOCS = Locus of Control Scale; PSSS = Perceived Social Support Scale; BDI = Beck Depression Inventory; MOCI = Maudsley Obsessional-Compulsive Inventory; BSI = Brief Symptom Inventory.

Otherwise, the correlations for general causality orientations, locus of control, perceived social support, depressive symptoms, psychological symptoms, and obsessive-compulsive symptoms are presented in Table 3.

**Table 3:** Correlation Matrix of Research Variables

Variable	1	2	3	4	5	6	7	8	9	10	11	12
1. GCOS- impersonal	-											
2. GCOS-control	.19**	-										
3. GCOS- autonomy	-.13**	.32**	-									
4. MOCI	.30**	.20**	-.06	-								
5. LOCS-total	.25**	.06	-.18**	.23**	-							
6. LOCS- external	.26**	.23**	-.07	.28**	.79**	-						
7. LOCS- internal	.13**	-.16**	-.27**	.00	.70**	.21**	-					
8. BSI	.38**	.13**	.11**	.46**	.25**	.34**	.04	-				
9. PSSS-family	.18**	-.03	-.17**	.11**	.11**	.12**	.05	.30**	-			
10. PSSS-friends	.19**	-.06	-.27**	.11**	.13**	.01**	.13**	.25**	.41**	-		
11. PSSS	.22**	-.05	-.24**	.12**	.14**	.13**	.09**	.33**	.91**	.74**	-	
12. BDI	.34**	.04	-.17**	.40**	.23**	.27**	.09**	.68**	.32**	.29**	.23**	-

*Note.* GCOS = General Causality Orientations Scales; GCOS- impersonal = Impersonal Orientations; GCOS-control = Control Orientations; GCOS- autonomy = Autonomous Orientations; MOCI = Maudsley Obsessional-Compulsive Inventory; LOCS = Locus of Control Scale; BSI = Brief Symptom Inventory; PSSS = Perceived Social Support Scale; BDI = Beck Depression Inventory.

*p < .05. **p < .01.

In the first model based on GCOS, three indicator variables (autonomous, control and impersonal orientation) were constructed using the parcel method based on exploratory factor analysis to show the latent variable (see Hall et al., 1999 for more information on the parcel method). In the second model on which the locus of control is based, two indicator variables (internal and external locus of control) were constructed using the parcel method based on exploratory factor analysis to illustrate latent variables. Analyzes were made using the maximum likelihood method and based on the model testing. When assessing compliance indices, it was used in the compilation of Hooper, Coughlan, and Mullen (2008). Analyzes were made using structural equation models.

Baron and Kenny (1986) proposed a set of concept to be examined for analysis of mediating role; which was used to investigate the mediating role of perceived social support in the relationship between locus of control and general causality orientations with psychological symptoms. Baron and Kenny (1986) laid out 4 step requirements that must be met to form a true mediation relationship. Accordingly, whether a variable act as a mediator variable depends on whether it encounters a four-digit list of criteria. A number of regression analysis and Sobel tests are required to test these criteria. Using this method, path analysis was conducted to ascertain the mediating role of perceived social support in the relationship between locus of control and general causality orientations with psychological symptoms (Depressive symptoms, OCD symptomology, and general psychological symptoms). Detailed information of the analysis can be seen in Table 4, Results of the analysis for male and female can also be seen in Table 5.

**Table 4:** Path Analysis Summary of General Causality Orientations and Locus of Control

Mediator (PSSS) Variable: Sub-dimension	Effect of mediator variable on predicted variable (Psychological Symptoms)					
	Depressive symptoms		OCD symptomology		General Psychological Symptoms	
	Sobel test	Total variance	Sobel test	Total variance	Sobel test	Total variance
General Causality Orientation						
Autonomy orientation						
Family support	-4.15**	Partial moderating (14%)	-2.46**	Partial moderating (10%)	-3.95**	Partial moderating (10%)
Friend support	-5.65**	Partial moderating (18%)	-2.79**	Partial moderating (10%)	-5.54**	Partial moderating (6%)
Control orientation						
Family support	-0.71	None	-0.69	None	-0.79	None
Friend support	-1.93*	Partial moderating (3%)	-1.65	None	-1.92*	Partial moderating (3%)
Impersonal orientation						
Family support	4.66**	Partial moderating (13%)	2.55**	Partial moderating (11%)	4.38**	Partial moderating (15%)
Friend support	4.51**	Partial moderating (14%)	2.61*	Partial moderating (11%)	4.41*	Partial moderating (12%)
Locus of Control						
Internal locus of control						
Family support	3.04**	Partial moderating (8%)	2.16*	Partial moderating (6%)	2.96**	Partial moderating (8%)
Friend support	1.96*	Partial moderating (4%)	1.69	None	1.99*	Partial moderating (4%)
External locus of control						
Family support	1.31	None	1.21	None	1.31	None
Friend support	3.38**	Partial moderating (9%)	2.33**	Partial moderating (7%)	3.31**	Partial moderating (10%)

*p < .05. **p < .01.

**Table 5:** Summary of Path Analysis for General Causality Orientations and Locus of Control by Gender

Predicting variable	Depressive symptoms		OCD symptomology		General Psychological Symptoms	
	Sobel test	Explanation	Sobel test	Explanation	Sobel test	Explanation
General Causality Orientation: Perceived Social Support From Family						
Female						
Autonomy	-3.09**	Partial moderating	-1.97**	Partial moderating	-3.01**	Partial moderating
Control	-0.24	None	0.25	None	-0.24	None
Impersonal	2.81**	Partial moderating	2.17*	Partial moderating	3.86**	Partial moderating
Male						
Autonomy	-1.96*	Partial moderating	-1.47	None	1.92*	Partial moderating
Control	-1.04	None	-0.95	None	-1.45	None
Impersonal	2.14*	Partial moderating	1.54	None	2.08*	Partial moderating
General Causality Orientation: Perceived Social Support From Friends						
Female						
Autonomy	-5.00**	Partial moderating	-2.16*	Partial moderating	-4.14**	Partial moderating
Control	-1.49	None	-1.28	None	-1.48	None
Impersonal	3.28**	Partial moderating	1.98*	Partial moderating	3.17**	Partial moderating
Male						
Autonomy	-3.15**	Partial moderating	-2.22**	Partial moderating	-2.90**	Partial moderating
Control	-0.98	None	-0.94	None	-0.96	None
Impersonal	3.13**	Partial moderating	2.21*	Partial moderating	3.13**	Partial moderating
Locus of Control: Perceived Social Support From Family						
Female						
Internal locus of control	3.07**	Partial moderating	1.98*	Partial moderating	2.98**	Partial moderating
External locus of control	1.54	None	1.31	None	1.52	None
Male						
Internal locus of control	1.04	None	0.94	None	1.03	None
External locus of control	1.56	None	1.28	None	1.54	None
Locus of Control: Perceived Social Support From Friends						
Female						
Internal locus of control	2.06	None	1.58	None	2.03	None
External locus of control	2.68	None	1.89	None	2.62**	Partial moderating
Male						
Internal locus of control	1.67*	Partial moderating	1.47	None	1.63	None
External locus of control	2.41**	Partial moderating	1.97*	Partial moderating	2.29**	Partial moderating

*p < .05. **p < .01.

**Figure 1:**
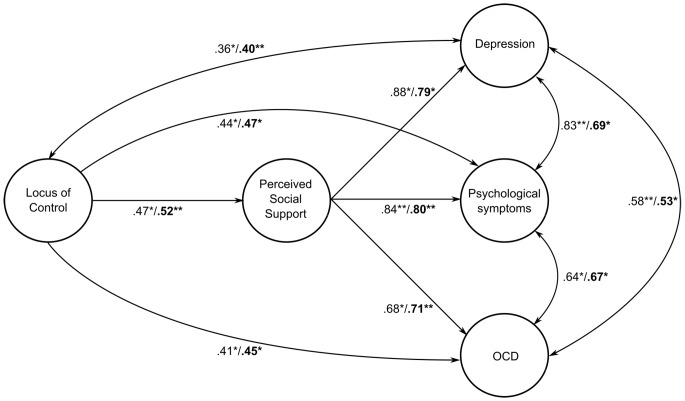
The Mediating Role of Perceived Social Support in the Relationship Between Locus of Control and Psychological Symptoms. N.B: While emboldened significance values show the significant relations of males, other, non-emboldened values display the significant relationships for females.

According to the analysis, the measurement model was found to have adequate fit indices, resulting in no need for correction. In this case, the identical structural model and measurement overlap. In relation to the tested structural models, the relationships between locus of control and overall psychological symptoms, OCD symptomology, and depressive symptoms were tested with AMOS 23, and the explained variance values are shown in Figure 1. When the model solely was tested for females, a fit index (χ^2^(27, *N* = 558) = 569.059, *p* < .001, GFI = 0.94, AGFI = 0.93, NFI = 0.90, CFI = 0.92, RMSA = 0.066). The tested structural model’s relationship in terms of locus of control for males is shown in Figure 1. When the model alone is tested, it was found to have a fit index (χ^2^(64, *N* = 192) = 325.667, *p* < .001, GFI = 0.97, AGFI = 0.94, NFI = 0.84, CFI = 0.91, RMSA = 0.071) for psychological symptoms (Depressive symptoms, OCD symptomology, and general psychological symptoms). Fit indices of the measurement model for locus of control and psychological symptoms (Depressive symptoms, OCD symptomology, and general causality orientations) are shown in Table 6, for females and males separately.

**Table 6:** Index of Compliance Measured by Structural Equation Modeling (N = 750, female = 558, male = 192)

Predicted variable	χ^2^	GFI	AGFI	NFI	CFI	RMSEA
Locus of control						
Female						
Depression	χ^2^(38, *N* = 558) = 178.982	0.96	0.94	0.94	0.95	0.066
OCD	χ^2^(38, *N* = 558) = 237.885	0.97	0.95	0.84	0.86	0.080
General psychological symptoms	χ^2^(33, *N* = 558) = 125.532	0.97	0.95	0.94	0.96	0.078
Whole Model	χ^2^(27, *N* = 558) = 569.059	0.94	0.93	0.90	0.92	0.066
Male						
Depression	χ^2^(32, *N* = 192) = 112.974	0.89	0.86	0.83	0.80	0.086
OCD	χ^2^(38, *N* = 192) = 122.488	0.92	0.91	0.82	0.88	0.084
General psychological symptoms	χ^2^(38, *N* = 192) = 126.191	0.95	0.94	0.87	0.92	0.086
Whole Model	χ^2^(64, *N* = 192) = 325.667	0.97	0.94	0.84	0.91	0.071
General causality orientations						
Female						
Depression	χ^2^(16, *N* = 558) = 52.797	0.98	0.96	0.97	0.98	0.051
OCD	χ^2^(28, *N* = 558) = 69.719	0.96	0.94	0.94	0.96	0.052
General psychological symptoms	χ^2^(31, *N* = 558) = 113.436	0.97	0.95	0.96	0.97	0.069
Whole Model	χ^2^(27, *N* = 558) = 469.987	0.96	0.93	0.91	0.94	0.069
Male						
Depression	χ^2^(18, *N* = 192) = 53.571	0.97	0.95	0.98	0.96	0.059
OCD	χ^2^(29, *N* = 192) = 62.129	0.96	0.94	0.98	0.97	0.061
General psychological symptoms	χ^2^(33, *N* = 192) = 80.425	0.94	0.93	0.91	0.94	0.087
Whole Model	χ^2^(30, *N* = 192) = 469.987	0.97	0.94	0.92	0.94	0.078

*Note.* For all χ^2^ tests *p* < .001.

**Figure 2:**
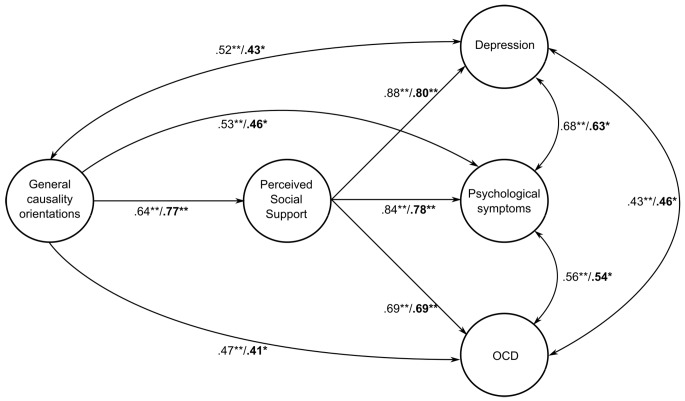
The Mediating Role of Perceived Social Support in the Relationship Between General Causality Orientations and Psychological Symptoms. N.B: While emboldened significance values show the significant relations of males, other, non-emboldened values display the significant relationships for female.

According to the tested structural models, the relationships between general causality orientations and overall psychological symptoms, OCD symptomology, and Depressive symptoms were tested with AMOS 23 and the explained variance values shown in Figure 2. When the model alone is tested, it was found to have a fit index (χ^2^(27, *N* = 558) = 469.987, *p* < .001, GFI = 0.96, AGFI = 0.93, NFI = 0.91, FCI = 0.94, RMSA = 0.069). The tested structural model’s relationships between general causality orientations and overall psychological symptoms, OCD symptomology, and depressive symptoms were tested with AMOS 23 and the explained variance values for males are shown in Figure 2. When the model is examined in relation to psychological symptoms, it was found to have a fit index (χ^2^ = 80.325, *SD* = 33, *p* < .001). Given the significant value (χ^2^(30, *N* = 192) = 469,987, *p* < .001, GFI = 0.97, AGFI = 0.94, NFI = 0.92, FCI = 0.94, RMSA = 0.078). Fit indices of the measurement model for general causality orientations and psychological symptoms (Depressive symptoms, OCD symptomology, and general causality orientations) are shown in Table 6 for females and males separately.

According to our results, when perceived social support from family has a partial mediating role in the relationship between autonomous orientation, impersonal orientation, and psychological symptoms (general psychological symptoms, OCD symptomology, and depressive symptoms), control orientations do not play a significant role in any psychological symptoms. The variance explained by the mediating role of perceived social support from family ranges from 10% to 15% When perceived social support from friends has a partial mediating role in the relationship between autonomous orientation, impersonal orientation, and psychological symptoms (general psychological symptoms, OCD symptomology and depressive symptoms), the variance explained by the mediating role of perceived social support from friends ranges from 3% to 18%. However, while perceived social support from friends has a partial mediating role in the relationship between control orientation and depressive symptoms, general psychological symptoms have a partial mediating role. On the other hand, it does not have a mediating role in the relationship between control orientations and OCD symptomology.

According to the results, when perceived social support from family has a partial mediating role in the relationship between internal locus of control and psychological symptoms (general psychological symptoms, OCD symptomology, and depressive symptoms), external locus of control does not have a significant role in any psychological symptoms. The variance explained by the mediating role of perceived social support from family ranges from 4% to 8%. When perceived social support from friends has a partial mediating role in the relationship between internal locus of control, depressive symptoms, and general psychological symptoms, but it does not have any mediating roles in external locus of control and OCD symptomology. As a partial mediator, perceived social support from friends has a role in the relationship between external locus of control and psychological symptoms. The explained variance of perceived social support from friends as a mediator ranges from 4% to 10%.

Analysis among both genders revealed that while perceived social support from family plays a partial role in respect to the relationships of internal locus of control with OCD symptomology, depressive symptoms, and general psychological symptom levels for female, in the case of support received from friends, locus of control has taken solely a partial role in depressive symptoms and general psychological symptoms. Moreover, friends' support data did not show any significant relationships with OCD symptomology. When it comes to female, perceived social support from family has no significant mediating roles relating to external locus of control; but as a mediator, perceived social support from friends has a partial mediating role in the relationship between external locus of control and general psychosocial symptoms. In relation to men, perceived social support from family had no mediating role in the relationships of internal locus of control; however, as a mediator, perceived social support from friends has a partial mediating role in the relationship between internal locus of control and depressive symptoms. On the other hand, perceived social support from friends has a partial mediating role in the relationship between external locus of control and depressive symptoms, OCD symptomology, and general psychological symptoms.

## Discussion

In our study, the relationship between general causality orientations and locus of control with psychological symptoms was analyzed while perceived social support was accounted as partial mediator. This kind of model has been studied for the first time in Turkey. The confusion about general causality orientations and locus of control, which has been discussed in the literature extensively (Soenens et al., 2005; Vansteenkiste & Sheldon, 2006), was clarified in our study. To underline the distinctive properties between these variables, each variable was analyzed separately and collectively as a partial mediator (Figure 1 and 2), and those analyses provided valuable insights about this distinction.

The proportions of female and male participating in the study are not equal. In this respect, data was sub-categorized according to gender and displayed separately. Analysis via structural equation modeling provided very well fitting indexes for both genders. Data obtained in this study was first analyzed according to general causality orientations, then locus of control variables have been discussed in respect to sub-categories of social support; support of family and friends. Moreover, the impact of culture was also evaluated in our study.

Obtained data about general causality orientations in this study support and confirm the previous findings of Deci and Ryan (1985). In both studies, autonomous individuals showed a reduced tendency to experience psychological symptoms. Moreover, the finding of an increased tendency toward psychological symptoms in impersonal individuals is also consistent with previous literature (Deci & Ryan, 1987, 2008; Brockelman, 2009; Sadabadi et al., 2011). In addition to this finding, social support, which has never been studied in this context as partial mediator, was demonstrated to decrease psychological symptoms in autonomous and impersonal individuals. However, social support was not completely unnoticeable in individuals who are control oriented. In control oriented individuals, the source of social support was proven to be important. While social support was not a partial mediator when received from family, support from friends significantly improved psychological well-being and decreased depressive symptoms. The reason behind this result might be owing to the escalation of distance between generations in nuclear-families (Kağıtçıbaşı, 1970), lack of communication and problem solving among family members (Cerit, 2007), increased clashes among family members during adolescence (Özgüven, 2001), and variations of support, interest, and communication of family depending on their socio-economic status (Genez Muluk, 2004; Kağıtçıbaşı et al., 2001; Ulusavaş, 1990). Moreover, since 84% of the individuals who participated in the study are bachelor students who are living with friends instead of family, the shift of social support from family to friends is considered to be an important factor about this finding (Hortaçsu et al., 1991; Lee, 2011).

The impact of social support for individuals with an internal or external locus of control on psychological symptoms was emphasized in our study. Support coming from family to individuals with internal locus of control exerted its effect on well-being by decreasing psychological symptoms. On the other hand, family support did not show any significant effect on individuals with external locus of control. This finding leads us to question whether social support from family is suitable as a partial mediator because there are several studies showing that individuals with an external locus have a higher tendency to experience psychological symptoms (Baydoğan & Dağ, 2008; Dağ, 1992; Daniels & Guppy, 1994; Erol et al., 2000; Ryff, 1989). These circumstances indicate that an individual's perception of their family’s social support directly regulates psychological status as a partial mediator. Moreover, the mediating roles of families' and friends' support between locus of control and psychological symptoms (depressive symptoms, OCD symptomology, and general psychological symptoms) was investigated via pathway analysis, and interpreted with reference to the previous literature. Although the theory is not in accordance with our study, we advise following studies to test alternative models in which the roles of variables are switched for mediator role. Sub-categorical analysis of gender showed that only females demonstrated significant partial mediating roles for family social support in relationship between locus of control and psychological symptoms. We think that this finding can be interpreted through the nature of internal locus of control. Internal locus of control combined with the beliefs of self-control might have diminished psychological symptoms (Dağ, 1992; Meijer et al., 2002). In traditional Turkish families, female’s beliefs about self-control and prolongation of well-being are thought to be highly correlated with families' social support. 

Cumulative analysis of families' social support revealed significant correlations between autonomy orientation, impersonal orientation, and internal locus of control with psychological symptoms. However, no significant correlation was found between control orientation and external locus of control with psychological symptoms. Despite significant correlations reported in western literature about general causality orientations and locus of control (Deci & Ryan, 1985, 1987; Williams et al., 1996), findings about impersonal orientations and external locus of control emphasize the importance of the distinction between these two personality types. Self Determination Theory, as it states initiation, regulation, and continuation of behavior are not only affected by external stimuli, but more importantly influenced from internal motivations. This provides important clues to clarify this our findings. Because impersonal orientation is affected by both consequences of behaviors and motivation (Deci & Ryan, 1985; Hagger & Chatzisarantis, 2011; Olesen, 2011; Olesen, Thomsen, Schnieber, & Tønnesvang, 2010). Especially, the conclusion, that external locus of control, which emphasizing external stimuli (social support) and showing no significant correlation, and impersonal orientations, pointing out the importance of motivation and internal stimuli and demonstrate significant correlation of interactive connection between internal and external processes. Moreover, the validated contributions of family support might be based on motivation triggered by external processes (Deci & Ryan, 1985; Koestner & Zuckerman, 1994). Discouraging children’s independence is frequently observed in families in Turkey, and more seriously, the independent personal development of children does seem to pose a threat to families (Kağıtçıbaşı, 1984). Owing to these phenomena, families will try to raise an obedient child having strong ties to family, even when they settle to a new location. However, this kind of parenting does not guarantee a strong attachment to family. As Kağıtçıbaşı (1970) stated, acting scrutinizing behaviors toward children cannot be accounted as loving or being supportive in Turkish family structure and these two notions are totally separate from each other. Especially for girls in Turkey, this securative behaviors show themselves as conservative and in Turkish culture, this effect is more noticeable compared to boys. Data obtained in this study also strongly support these notions.

Social support coming from friends has shown significant effects on the relationships between autonomous and impersonal orientations and depressive symptoms, OCD symptomology, and general psychological symptoms. However, the impact of social support was not similar to control oriented individuals. Those individuals experienced increased general psychological symptoms and depressive symptoms, but not OCD symptomology. Control orientation was not significant for both gender. An important feature of the GCOS is that each subscale does not sub-categorize or separate individuals. This lies in the nature of general causality orientations being more dimensional than categorical. At each subscale, rather than a separation, each individual should be considered to take a place on an axis; to illustrate, control oriented individuals should be considered and analyzed on an autonomous and impersonal axis (Deponte, 2004). The investigation of individuals with an internal or external locus of control (with the partial mediator of friends’ social support) disclose significant relationships between internal locus of control, depressive symptoms, and psychological symptoms, but not with OCD symptomology. Moreover, gender-based analysis illustrated significant relationships between internal locus of control and psychological symptoms solely in males. This kind of difference is thought to be the result of variations in sub-dimensions of locus of control. In the literature, there are discussions about variations in sub-dimensions of control individuals, and they review the expansion of female’s social networks and how they might have an impact on these variations (Sherman et al., 1997). However, since we investigated internal and external locus of control in two dimensions only, this kind of analysis could not be inferred from our study. It is well known that individuals at university level initiate distant relations with family, at which point perceived support shifts from family to friends (Lee, 2011). Our study supports this notion but it does not explain the gender difference.

Our study illustrates that suitable independent variables for depressive symptoms are impersonal orientation, autonomous orientation, and external locus of control. While increasing impersonal orientation and locus of control causes elevated depressive symptoms levels; increased autonomous orientation related to declining depressive symptoms levels. Moreover, when social support is accounted for as a partial mediator, the perceived social supports showed distinct results. Perceived support coming from friends was shown to be much more effective in reducing depressive symptoms levels, rather than family. Previous research on the topic shows that students who are living far away from family require more psychological support (İmamoğlu & Yasak-Gültekin, 1993), and decreased family support is correlated with increased psychological symptoms (Armsden & Greenberg, 1987; Bayram, 1999; Wentzel, 1998). There are also some studies which point out the importance of family toward the progression of depressive symptoms (Chou, 1999; Juang & Silbereisen, 1999) while some other studies relate importance of elevated friends support on declined depressive symptoms (Wentzel, 1998). Moreover, Chou's (1999) research showed that friend’s support has independent effects on personal well-being and an increased number of friends are associated with positive emotions. Since all of these findings are covered in our study, new analyses were established, and those analyses revealed that the majority of students were senior university students (78%). Accordingly, our study showed that junior and senior university students give more importance to friends than freshman (sophomore) university students. Previous studies of depressive symptoms that describe social support as an important factor for treatment, showed that lack of perceived social support is recognized as an initiator and maintenance factor for depressive symptoms, and found that depressive symptoms increase without social support (Paykel, 1994). Moreover, perceived social support was found to be critical for depressive symptoms treatment in the early phases (Brugha et al., 1997). In our study, most of the participants were far away from their hometown and living with friends in other cities. Especially, while assessing the risk factors and managing protective measures for psychological well-being of freshman and sophomore students, different effects of perceived social support should not be neglected and must be implemented as critical factors.

Suitable independent variables for OCD symptomology were impersonal orientations, control orientation, and external locus of control. An increase in each of these variables was associated with elevated OCD symptomology levels. While social support has partial mediator effects, symptoms for both impersonal orientation and external locus of control were decreased. However, this effect has not been observed for OCD symptomology when control orientation has a mediating role, and neither perceived support of family nor friends has an effect on OCD symptomology levels. Control orientation was studied as an independent variable while conducting pathway analysis combined with literature. However, another study (Neighbors et al., 2006) in which these variables were in a different order and different theories were tested, showed that control orientation has significant contributions as a partial mediator. We advise to future studies to include control orientation as a partial mediator as well. When perceived social support is considered as a partial mediator, the source of support is proven to be important. While perceived social support is a partial mediator, and received from family, individuals having an external locus of control experienced reduced OCD symptomology Although this situation might have arisen because of the probable co-existence of depressive symptoms and OCD symptomology (Andrews et al., 2002; Pigott et al., 1994), it might have happened due to the impact of depressive symptoms on cognitive functions, leading to vulnerability for OCD symptomology, or statistically masking symptoms of depressive symptoms toward OCD symptomology (Rachman, 1998).

According to the findings of our study, the most suitable independent variables for general psychological symptoms are impersonal orientations and external locus of control. In most cases where both perceived support from family and friends are partial mediators, a significant decrease in psychological symptoms was observed. Only in the situation where social support from family is a partial mediator, reduced psychological symptoms were detected in individuals having an external locus of control. In our study, the most applicable dependent variables are depressive symptoms, general psychological symptoms level, and OCD symptomology, respectively.

### Conclusion

There were some limitations in our study, namely the fact that all the data acquired in this study was based on self-reports. Although covering many students from different cities expands the breadth and replicability of the study, comprising only university students limits both age and educational levels, and results in a limited sample in Turkey. Therefore, the results obtained in terms of age and culture-dependent features can be mentioned. However, choosing university students who are in transition from puberty to adulthood is representative of an important part of society. In addition, these findings can better help us inform protective psychological measures for these individuals. However, involving other age groups in future studies will expand the knowledge about age group differences and their interpretations. Furthermore, since our study does not comprise clinical samples, the findings present limitations about their clinical use. On the other hand, our study focused more on testing models and differentiating theoretical variances across different cultural backgrounds, rather than clinical findings. It will be advantageous for future studies to include clinical samples.

Non-equal distribution of our sampling in regard to gender presents difficulties when analyzing gender specific differences. Owing to this factor, our study does not provide a comparative analysis between gender, and analyses for each gender were performed separately. Although the sample for male is tried to be expanded, because of in-equal number of individuals gender in departments and voluntary basis, this effort could not be completely accomplished. Including more male individuals will broaden the study and enable comparative studies about gender.

As expected from the literature, the established model was confirmed to be productive for undergraduate students in Turkey. Sub-dimensions of causality orientations and locus of control were analyzed in detail and different results from western culture were obtained. This difference was discussed and thought to be based on for undergraduate students in Turkey. Especially, gender and attitudes toward raising children are considered to be the main reasons for this difference. Perceived social support was found to be suited for the role of partial mediator, and social support from friends was found to have more positive roles than social support from family. As age, environment, and the distance from family were account for in this study, we were able to make critical deductions. Since other similar studies are focused on individualistic cultures, owing to mosaic nature of Turkey, our findings provided prosperity and variance to the literature, and reveal the variances of similar studies in different cultures. Finally, general causality orientations, locus of control, perceived social support acting as variables showing different psychological symptoms can be used in different models and can assume different partial mediating roles.
